# Public Management for Local Development: Perception from the Administrative Area of a Municipality in Peru

**DOI:** 10.12688/f1000research.143844.1

**Published:** 2024-02-19

**Authors:** Rosmery Sabina Pozo Enciso, Susana Marleni Atuncar Deza, Oscar Arbieto Mamani, Miguel Gerardo Mendoza Vargas, Hilda Luzmila Felix Pachas

**Affiliations:** 1 Universidad Autonoma de Ica, Chincha Alta, Ica, Peru; 2 Universidad Nacional Micaela Bastidas de Apurimac, Abancay, Apurimac, Peru; 3 Universidad La Salle, Pedro Vilcapaza, Arequipa, Peru

**Keywords:** Public management, state entity, administration, resources.

## Abstract

**Objective:**

To analyze the perception of the administrative department of a municipality regarding public management.

**Method:**

Qualitative approach through interviews. The sample consisted of 30 employees from the administrative department of a municipality in Peru.

**Results:**

It was found that the employees seem to have a clear understanding of the applied public management model and the organization of the administrative area. Responses regarding the management of financial, human, and material resources vary in accuracy and detail, suggesting a possible lack of knowledge in some areas. It is important to enhance communication clarity on these topics and provide precise information to ensure effective and transparent management within the municipality.

**Conclusion:**

Municipality employees demonstrate an understanding of their roles and face challenges in teamwork and bureaucracy. There is some confusion regarding the “New Public Management” and a lack of knowledge in resource management. Improving training, streamlining procedures, and fostering team participation in planning are crucial aspects. Furthermore, implementing periodic assessments is recommended to enhance administrative efficiency.

## Introduction

The term or concept of public management refers to the planning, organization, direction, and control of various activities carried out by the public sector, with the aim of achieving the objectives and goals set by governmental institutions ( Cañari & Hancco, 2021). In other words, public management involves efficiently administering public resources, both in decision-making and implementation, with the purpose of enhancing the societal benefits for the entire community ( Morveli, 2021).

Given the above, it can be inferred that poor public management by governmental entities can lead to various problems and challenges that can affect both internal operations of the organizations and the well-being and trust of the general population ( Merchán et al., 2022).

Globally, the advent of COVID-19 in 2020 not only impacted the economic and political landscape of countries but also unveiled significant issues in public management on a global scale. Over the two years since the onset of the coronavirus pandemic, countries have required not only effective preventive measures and treatments but also targeted and efficient public policies to address the public health emergency. The pandemic has posed a threat to existing governance systems, highlighting issues of management, leadership, and the limitations of modern democracy in various parts of the world ( Assefa et al., 2022).

Within this framework, it’s crucial to understand that public management is a significant factor in society. Hence, it’s essential to comprehend how a state institution behaves and how it assembles different strategic elements to tackle challenges posed by both external and internal agents within its jurisdiction ( Ramió, 2020). The upper management should implement proper strategic planning to ensure the institution’s competitiveness and adaptability to changes. Sound management ensures the continuity and longevity of institutions. On the contrary, poor management will have negative impacts not only within the institution, affecting employees and resulting in low motivation and performance but also within the society in which the institution operates ( Correia et al., 2019).

Arévalo and Barbarán (2021) noted that corruption is a significant issue plaguing Peru, stating that it is “one of the main concerns of Peruvians about the performance of both the State and the Government. One option to combat corruption is to foster transparency mechanisms in the administration of public resources. Public officials are called to serve the interests of the Nation and to ensure the proper use of public resources transparently” (p. 5526). While corruption is a major concern in Peru, it’s also apparent that authorities often lack the support of the population. In Chiclayo, for instance, 69% of citizens feel that the authorities of the municipal district of José Leonardo Ortiz (Chiclayo) do not support them, and 84% indicate that they don’t believe the municipality considers the population’s demands. This is a clear example that local authorities are not effectively practicing public management, as reflected in the opinions of the residents ( Mallasca et al., 2018).

While public opinion is essential, so is the perspective of those working within public institutions. Public employees often find themselves entangled in the problems faced by the entity, due to bureaucratic obstacles and the complexity of administrative procedures within municipal areas. This often leads to inefficiencies in public management models, administrative processes, and procedures, or lack of planning that result in delays or hindrances to local development. Therefore, this research aims to understand how public management is conducted within a municipality from the viewpoint and perception of administrative staff.

## Methods

The research will employ a group interview approach with a qualitative focus ( Hernández-Sampieri et al., 2018). The research design will be phenomenological and descriptive. This is because the researcher will use interviews to collect information regarding experiences and opinions of the sample, which could be diverse or common ( Hernández-Sampieri et al., 2018).

The population will consist of 100 employees from the administrative area of a municipality in Peru ( Ñaupas et al., 2018). The sample will comprise 30 administrative staff members from a municipality. The specific job titles held within the institution will not be considered; the criterion is that they occupy purely administrative roles within the study site.

The chosen sample size is based on the nature of the study, which aligns with qualitative research principles. “Sample sizes for qualitative studies, such as grounded theory, interview studies, or observations of people, suggest a sample size of 20 to 30 cases to study” ( Hernández-Sampieri & Mendoza, 2018, p. 428).

It’s important to note that qualitative studies, such as interviews or observations, do not seek to generalize results. The researcher’s focus in such studies is on the depth of the topic. Selecting the sample to study involves considering three factors: the researcher’s operational capacity, the unit of analysis’s knowledge of the phenomenon under study, and the time available for data collection. Furthermore, the sample will be composed of “typical cases,” aiming for rich, in-depth, and high-quality information, without emphasizing quantity or standardization ( Hernández-Sampieri & Mendoza, 2018).

Considering the above, no specific sampling method will be used.

The technique employed was the interview, and the instrument used was an “interview guide.” This guide was applied between the months of May and June 2023. Participants were previously notified by e-mail. A recording was made of the responses obtained from the interview in order to be able to analyse the responses in depth.

The researcher respected all aspects related to copyright while conducting the research. Each participant was adequately informed about the research, and their participation was completely voluntary and willing. Anonymity of the interviewees was maintained, and the confidentiality of obtained data was upheld. No modifications were made to the collected responses.

### Ethics statement

The research was approved by document CO-001-06-2023/CE issued by the ethics committee of the Universidad Autonoma de Ica.

### Ethical aspects

The confidentiality and privacy of participants has been ensured in accordance with the ethical principles of confidentiality, respect and transparency. Measures were taken to preserve the identity of the interviewees by means of pseudonyms or codes for anonymity in the results. Participation was voluntary, with written informed consent, detailing objectives and possible implications. A relationship of respect and empathy was maintained, creating an environment of trust for the free expression of experiences. Participants were free to withdraw at any time without adverse consequences.

## Results

Regarding gender, most individuals within the sample were women, accounting for 71.4% of the participants in the study, while men constituted 28.6%. In terms of age distribution, 33% fell within the 20-25 age range, 37% were aged 26-35, and 30% fell between the ages of 36 and 55.

Moving on to the dimension of administrative workers’ backgrounds, it was observed that most participants were aware of their roles within the municipality. Roles mentioned included administration, administrative assistants, handling paperwork, engineering/technical roles, human resources, and specific responsibilities such as being a manager, tax area supervisor, warehouse manager, logistics coordinator, cashier, among others. Regarding their tenure in these roles, it was found that some had been in their positions for less than six months, others for around 6 months to a year, and some had experience ranging from 2 to 8 years. Some participants mentioned lacking experience or having worked in different fields unrelated to public management. The shortest work experience period mentioned was “3 months,” cited by one participant, while the longest was 8 years.

When it comes to the challenges perceived by workers within the municipality, teamwork was a prominent concern. Some considered managing a large team to be the primary challenge, which could complicate internal coordination and communication. Therefore, they felt compelled to apply effective strategies to ensure efficient and smooth management. Another challenge identified was project process control and monitoring, which are crucial elements of public management. Participants indicated that inadequate supervision could impact the achievement of objectives. Excessive bureaucratic procedures and lengthy administrative timelines were also mentioned as daily challenges hindering efficiency among departments and the management of public resources. This often resulted in delays in response times between departments and to the public. Lack of training in certain areas, such as logistics, was recognized as a challenge for employee development and effective job performance. This issue stemmed from the designation of unqualified personnel for certain roles, leading to performance and efficiency problems in the administrative area.

In terms of the public management model indicator, a considerable number of responses mentioned that the municipality adhered to the “New Public Management” approach as the model for promoting local development. However, some indicated that the “New Public Management” was being implemented based on results. Others perceived a more traditional management approach. There seems to be some confusion and lack of clarity regarding the exact model being implemented by the municipality. Nevertheless, a general sense of ongoing development and implementation of public management was apparent.

Regarding their understanding of the hierarchical organization of both their department and the municipality, responses indicated a clear consensus that the highest authority was the mayor, alongside the overseeing managers. The hierarchical structure mentioned in the responses ranged from the mayor and municipal council as the highest authorities, followed by managers and department heads in various areas. When asked about the management of financial, human, and material resources within the administrative area, responses varied, suggesting a lack of understanding in this regard. Inconsistent and unclear responses indicated a possible lack of information or comprehension regarding resource management within the administrative area of the municipality.

Concerning the administrative processes and procedures indicator, responses regarding “Key Administrative Processes and Procedures” showed significant differences. Many considered process supervision and control as key points, along with training and delegation of tasks. However, some responses indicated that a key process for improving municipality management could be the reduction of bureaucracy and streamlining of documentation. These management processes could enhance institution functionality. To improve process efficiency, many emphasized the importance of continuous employee training, which would enable the utilization of various technologies and platforms to streamline municipality management.

When asked about measures taken to ensure efficiency and transparency in resource management, a wide range of approaches were mentioned. Some highlighted the importance of project planning and periodic evaluation, while others emphasized the need for consensus-based and responsible decision-making. Citizen participation was also mentioned as a means of ensuring transparency in management.

Regarding the planning and sustainability indicator, opinions regarding the preparation and execution of strategic plans and development within the municipality highlighted the formulation of short- and long-term plans and goals with execution timelines. Inclusion of the entire team in the planning process was considered vital for clarity and focus during execution. For ensuring project and program sustainability, continuous supervision and monitoring were deemed crucial to ensure proper infrastructure utilization. Being adequately prepared and trained to maintain programs and projects over time was also emphasized.

When questioned about potential actions for implementing improvements and optimizing administrative processes, responses mentioned identifying areas for improvement, setting objectives, planning, and continuous monitoring. The significance of personnel training and continuous process review was highlighted. Some mentioned the implementation of internal directives and coordination among management areas.

Finally, in terms of evaluation and continuous improvement, performance and results within the administrative area were measured using internal indicators and tracking, as well as results-based evaluations. Internal surveys and evaluations conducted periodically were also emphasized. Some mentioned that they had not undergone performance evaluations during their time at the municipality but considered it necessary for improving processes through feedback. Potential actions for implementing improvements and optimizing administrative processes included identifying areas for enhancement, formulating objectives, and continuous planning and monitoring. Training staff and ongoing process review were also highlighted. Key indicators for measuring the success and efficiency of the administrative area included resource utilization, service quality, and the final outcomes of both short- and long-term plans.

## Discussion

In relation to the indicator of administrative worker backgrounds, the personnel conveyed that they are aware of and understand their roles within the municipality. They also mentioned the challenges they perceive within the municipality and each specific work area. This is significant as some participants mentioned that this is their first experience in public sector management. Despite the lack of experience in some cases, having a clear understanding of their tasks and responsibilities indicates that topics like control and supervision are emphasized within the institution. Nonetheless, it’s important to note that this is not always the case, as demonstrated by Luna’s study (2021) which found that “most of the workers’ responses were questions, stemming from lack of knowledge and task/function discoordination due to absence of leadership from superiors.”

Regarding the administrative processes and procedures indicator, responses varied. Workers indicated that key points followed by the municipality include planning, periodic project evaluations, and process control. They suggested consensus-based decision-making and citizen participation to ensure transparency and efficiency. These highlighted points and recommendations, from the workers’ perspective, contribute to appropriate and correct public management within the locality. Poor management can result in what Luna (2021) concluded: “there was a level of management with deficiencies due to lack of planning, direction, and strategies, resulting in failure to achieve planned objectives.”

In relation to planning and sustainability, it was evident that workers consider it crucial to include the entire team in the development and execution of strategic and development plans within the municipality. Continuous supervision is also considered a measure to ensure project and program sustainability. Similar findings were reported by Auccatinco and Auccatinco (2021), whose study revealed that all municipal workers in their research agreed that proper internal control and accountability positively affect development not only within the municipality but also in ongoing and upcoming projects.

In terms of the evaluation and continuous improvement indicator, workers indicated that the administrative area is measured using internal indicators and tracking, as well as through surveys and periodic evaluations. Key indicators of success and efficiency in the administrative area include resource utilization, service quality, and the final outcomes of both short- and long-term plans. These are important points that must be managed within any government institution. This aligns with the findings of Bautista and Delgado (2020), whose study evidenced challenges faced by administrative staff in a municipality during the COVID-19 pandemic. They reported issues such as a lack of activity planning, lengthy administrative processes, bureaucratic obstacles, and deficient communication among departments.

In summary, the perceived model of public management among the workers is mainly aligned with the “New Public Management” approach. While responses reflect a mix of understanding and lack of knowledge on some topics, some workers seem to have a clear grasp of the applied public management model and the organization of the administrative area. Although certain areas (such as the management of financial, human, and material resources) reveal confusion or a lack of information, the overall perception of workers appears positive. However, they also acknowledge areas that need improvement to optimize management and contribute to local development. These findings are consistent with Vega’s study (2019), which provided insight into the perception of management within a municipality and revealed that 94% of respondents considered the municipality’s management effective.

## Conclusion

In conclusion, the majority demonstrated an understanding of their roles within the municipality, encompassing various functions and specific responsibilities. Furthermore, a variety of work experiences in terms of tenure in positions were evident. The primary challenges identified by the workers in the municipality revolved around teamwork, process control, bureaucracy, extended administrative timelines, and a lack of training in certain areas.

While the perception that the municipality operates under the “New Public Management” prevails, certain confusions and lack of knowledge about the exact model were also observed. Regarding the management of financial, human, and material resources, a widespread lack of understanding was detected in the responses, suggesting the need to enhance transparency and clarity in this aspect. In the realm of administrative processes and procedures, diverse opinions on key points to improve municipal management were found, with a focus on the importance of continuous training and streamlining documentation. Concerning planning and sustainability, the necessity of involving the entire team in the development and execution of strategic plans was emphasized, as was the importance of constant oversight to ensure project sustainability.

Finally, in the indicator of evaluation and continuous improvement, the need for implementing periodic performance assessments to provide feedback and enhance administrative processes was identified.

## Author roles

Rosmery Sabina Pozo Enciso: Conceptualization, Formal Analysis, Investigation, Methodology, Supervision, Visualization and Writing – original draft

Susana Marleni Atuncar Deza: Methodology, Visualization, Formal Analysis and Writing – original draft preparation

Oscar Arbieto Mamani: Resources, Validation and Writing – Review & Editing

Miguel Gerardo Mendoza Vargas: Formal analysis, Visualization and Writing – Review & Editing

Hilda Luzmila Felix Pachas: Formal analysis, Visualization and Writing – Review & Editing

## Data Availability

Zenodo. Public Management for Local Development: Perception from the Administrative Area of a Municipality in Peru. https://doi.org/10.5281/zenodo.10511055. Data are available under the terms of the Creative Commons Zero v1.0 Universal (CC0 License).
