# Supplementary material for: Public Management for Local Development: Perception from the Administrative Area of a Municipality in Peru
Source: F1000Res. 2024 Feb 19;13:113. [Version 1] doi: 10.12688/f1000research.143844.1 (PMC11137478; doi:10.12688/f1000research.143844.1)
Supplement: Peer review history

# Reviewer report: Reviewer response for version 1

**DOI:** 10.5256/f1000research.157559.r248960

**Published:** 2024-05-29

**Authors:** Ambrocio Teodoro Esteves Pairazamán

**Affiliations:** 1 Facultad de Farmacia y Bioquímica, Universidad Privada Norbert Wiener, Lima, Peru

The manuscript explores municipal public management in Peru from the perspective of administrative staff, analyzing topics such as strategic planning, daily challenges, and the adoption of public management models. Based on surveys and qualitative analysis, it offers a detailed view of internal practices and critical areas for efficient and transparent local development.

 This article is highly relevant as it provides an insider’s perspective on public management in a Peruvian municipal context, identifying challenges and opportunities to improve administrative efficiency and transparency. It is particularly valuable for academics and professionals interested in public administration and local development.

 As a final recommendation, I would suggest that the institution implement periodic performance evaluations to provide feedback and improve administrative processes.

 I will keep an eye out for any new developments and their potential publication soon.

Is the work clearly and accurately presented and does it cite the current literature?

Yes

If applicable, is the statistical analysis and its interpretation appropriate?

Not applicable

Are all the source data underlying the results available to ensure full reproducibility?

Yes

Is the study design appropriate and is the work technically sound?

Yes

Are the conclusions drawn adequately supported by the results?

Yes

Are sufficient details of methods and analysis provided to allow replication by others?

Yes

Reviewer Expertise:

Master's degree in Research and Teaching, doctorate in Educational Administration, and Bachelor of Science in Management

I confirm that I have read this submission and believe that I have an appropriate level of expertise to confirm that it is of an acceptable scientific standard.

# Reviewer report: Reviewer response for version 1

**DOI:** 10.5256/f1000research.157559.r263191

**Published:** 2024-05-23

**Authors:** Juliana Abagsonema Abane

**Affiliations:** 1 Kwame Nkrumah University Science & Technology, Kumasi, Ghana

**REVIEW REPORT: PUBLIC MANAGEMENT FOR LOCAL DEVELOPMENT**

 The research topic is suitable for the study. The study employed a qualitative approach with interviews as stated in the methodology, and the sample size used was appropriate. However, the introduction of the paper needs improvement. The information provided on the topic is too scanty. The concept of “public management” has not been well explored in this study. Also, the right literature was not consulted to identify the theoretical and empirical debates on public management and local development to which the authors seek to contribute or provide the missing link. As a result, the paper lacks a clearly defined research puzzle/problem statement. 

 The study lacks a theoretical and empirical literature review. A section should be created for the review of existing relevant empirical literature on the topic to support the study. The review should be extant and of current literature focusing on different dimensions of public management and local development to provide a better understanding of public management. In the same way, an appropriate theory should be adopted to guide and ground this study. 

 Again, the methodology of the study fails to elaborate on exactly how the data collected using interviews were analyzed. The authors should provide a detailed account of how responses from the interview were analyzed. How were the interviews coded, and what qualitative data analysis method was employed? 

 The discussion section needs improvement. The findings of the study should be well discussed with the outcomes of earlier studies on the topic to identify where the findings of the current study confirm already existing literature or run contrary to the claims of earlier scholarship on the topic under investigation. 

 Finally, the conclusion provides a snapshot of the key findings of the study. Nevertheless, it is important to relate these findings to the possible implications. What is the implication of these observations on policy and practice in public management? What is the implication for future research on the topic?

Is the work clearly and accurately presented and does it cite the current literature?

Partly

If applicable, is the statistical analysis and its interpretation appropriate?

Not applicable

Are all the source data underlying the results available to ensure full reproducibility?

No source data required

Is the study design appropriate and is the work technically sound?

Partly

Are the conclusions drawn adequately supported by the results?

Partly

Are sufficient details of methods and analysis provided to allow replication by others?

No

Reviewer Expertise:

Research methodology, espcially, qualitative analysis. Also, my areas of research and expertise include public management and local government.

I confirm that I have read this submission and believe that I have an appropriate level of expertise to confirm that it is of an acceptable scientific standard, however I have significant reservations, as outlined above.

# Reviewer report: Reviewer response for version 1

**DOI:** 10.5256/f1000research.157559.r267976

**Published:** 2024-04-30

**Authors:** Kuno Schedler

**Affiliations:** 1 University of St. Gallen, St. Gallen, Switzerland

The general topic is topical and important, and it justifies a study on this scale. It is right that the authors use a qualitative method, as they give relatively high weight to the "how" and "why". Overall, I find the focus on Peru particularly interesting, as we in the English-speaking world know too little about public management in the LATAM countries.

 I would like to add a few points that could make the paper a little stronger overall:

 - I miss a focused research question. As it stands, the paper describes the respondents' answers, but it lacks an actual and in-depth analysis in relation to one (!) clear and focused question. For example, what does it mean when respondents see a number of challenges? What is the effect on services? Or for the acceptance by the population mentioned in the introduction?

 - In the description of the method, it would be important to know how the interviews were analyzed. Which coding method was used? With what results? I recommend using a paper that has been successfully published in a journal and contains qualitative evaluations as a guide. There are usually also good visualizations (e.g. with 1st and 2nd order analyses).

 - The discussion section should contain less of a summary of the results and more of a classification of your own findings in the context of existing knowledge. Which statements in the literature can be confirmed? Which ones need to be questioned? Where do the authors have new findings that cannot be found in the literature?

 Overall, I think that the paper can benefit if the focus is even narrower and if there is a concrete statement at the end. "So what?" - what do we learn from the analysis of the interviews? Beyond the descriptive, the analytical should be strengthened so that perhaps one or two surprising points come to light.

 I encourage the authors to continue working with this valuable and rich empirical material. We would like to see it published! The paper still needs some work, but it has the potential to be really exciting.

Is the work clearly and accurately presented and does it cite the current literature?

Partly

If applicable, is the statistical analysis and its interpretation appropriate?

Not applicable

Are all the source data underlying the results available to ensure full reproducibility?

No

Is the study design appropriate and is the work technically sound?

Partly

Are the conclusions drawn adequately supported by the results?

Yes

Are sufficient details of methods and analysis provided to allow replication by others?

Partly

Reviewer Expertise:

Public Management

I confirm that I have read this submission and believe that I have an appropriate level of expertise to confirm that it is of an acceptable scientific standard, however I have significant reservations, as outlined above.
